# High‐Resolution 3‐Dimensional Micro‐CT Imaging of Intervertebral Discs Using a Novel Contrast Agent

**DOI:** 10.1002/jsp2.70125

**Published:** 2025-10-07

**Authors:** Madison M. Buckles, Abdulkadir Nur, Nada H. Warda, Jacob C. Moncher, Mohammad Yunus Ansari

**Affiliations:** ^1^ Department of Biomedical Sciences College of Medicine, Northeast Ohio Medical University Rootstown Ohio USA; ^2^ UH Research Scholar Program Northeast Ohio Medical University Rootstown Ohio USA

**Keywords:** annulus fibrosus, contrast agent, contrast‐enhanced micro‐CT, intervertebral disc, intervertebral disc degeneration, Iohexol, low back pain, nucleus pulposus, potassium iodide, PTA

## Abstract

**Background:**

The nucleus pulposus (NP) in the intervertebral disc (IVD) is the first structure to exhibit degenerative changes during IVD degeneration (IDD). Currently, micro‐computed tomography (micro‐CT) imaging of the NP is a limiting factor in detecting IDD at an early stage. While contrast‐enhanced micro‐CT has been investigated, an effective contrast agent for IVD has not been identified. This study investigates potassium iodide (KI) as an effective contrast agent for micro‐CT‐based IVD visualization across multiple animal models to study IDD.

**Methods:**

We collected tails and spines from mice, rats, rabbits, sheep, and stained them with KI followed by micro‐CT imaging. For IDD, we performed caudal annular needle puncture surgery (NPS) in age and sex‐matched mice (*n* = 10) and stained with KI for imaging with micro‐CT. For the aging model, we compared IVDs from old to young mice.

**Results:**

Compared to unstained IVDs, KI effectively stained and visualized the 3D structure of the NP, exhibiting X‐ray attenuation properties comparable to bone. KI contrast staining enabled accurate and reproducible quantification of IVD height and NP volume. The cross‐sectional micro‐CT images of NPS IVDs were indistinguishable from the histological findings of the same sample and showed similar degenerative changes in the NP. We also found that KI staining is reversible, and the tissue remains compatible with downstream histological processing and immunostaining. Notably, KI successfully stained the NP in decalcified tissue, offering an advantage for NP analysis by removing bone background in micro‐CT scans. Additionally, we estimated that up to 15 sagittal sections, each 5 μm thick with 75 μm spacing, would be needed to fully assess IVD degeneration in mice.

**Conclusions:**

This study demonstrated that KI can be used to positively stain NP in the intact tail or spine and provide qualitative and quantitative data without any adverse effects on the immune/histological processing of the samples.

## Graphical abstract

This study introduces the first use of a contrast agent (potassium iodide) to enhance 3D imaging of the nucleus pulposus within an intact intervertebral disc (IVD). This method was used to demonstrate consistent 3D imaging of the nucleus pulposus across multiple animal models, including spine and tail IVDs. Additionally, this research confirms the method's effectiveness for studying intervertebral disc degeneration. This approach significantly improves our capacity to examine early‐stage nucleus pulposus degeneration in animal models before notable bone changes occur.

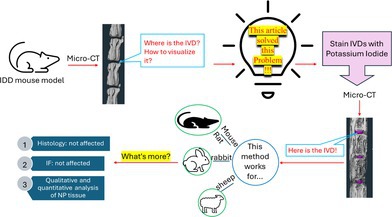

## 1 Introduction

The intervertebral disc (IVD) is a fibrocartilaginous structure between two adjacent vertebrae in the vertebral column, providing flexibility and dampening loads in the spine. The IVD consists of a central, proteoglycan‐rich, jelly‐like nucleus pulposus (NP) surrounded by lamellae of annulus fibrosus (AF), which is sandwiched between two vertebrae with a cartilaginous end plate (Figure S1) [1, 2]. The NP tissue plays a vital role in the function of the IVD, and changes in NP structure promote IVD degeneration and low back pain [3, 4, 5].

Intervertebral disc degeneration (IDD) is highly prevalent and a leading cause of low back pain (LBP), representing a significant socioeconomic burden costing billions of dollars in healthcare and lost productivity [4, 6, 7]. A better understanding of the mechanisms and structural and molecular changes in IVD during IDD is vital for developing therapeutic strategies for treating low back pain. One of the challenges associated with understanding the mechanisms of IDD is the lack of high‐resolution 3D imaging techniques to visualize the NP. Imaging by micro‐computed tomography (micro‐CT) relies on the attenuation of X‐rays passing through tissues up to the micrometer resolution limit; however, soft tissues such as IVDs have low X‐ray attenuation properties and are difficult to visualize without the help of a contrasting agent. Contrast‐enhanced micro‐CT is an important tool for studying the three‐dimensional microstructures of soft tissue, which otherwise have low X‐ray attenuation properties [8].

Small animal models, such as mice and rats, are of paramount importance for studying the mechanisms of disease pathogenesis due to the availability of genetically engineered animals and the feasibility of the work. However, current micro‐CT imaging to examine changes in the IVD is based on a negative staining approach where the NP is not visible, and changes in IVD height and other bone parameters are considered damage to the NP and overall IVD structure [9, 10, 11]. Many studies have shown that contrast‐enhancing agents such as Lugol's solution (Iodine‐potassium iodide), Hexabrix (ioxaglate), Cysto Conray II (iothalamate), CA^4+^, PMA (phosphomolybdic acid), and PTA (phosphotungstic acid) improve the visibility of soft tissues by micro‐CT [12, 13]. However, these approaches take a long time and preferentially stain the AF. In this study, we demonstrate a quick, positive staining approach of the NP by potassium iodide (KI), resulting in the visualization of the NP by micro‐CT. We show here that KI differentially stained the NP tissue in mouse and rat IVDs and enabled us to image the 3D structure of the NP at a 10 μm spatial resolution. Moreover, with this new method, we can quantify the volume of both a healthy and a degenerated NP in a mouse model. Here, we show that KI can be used as a contrast agent to visualize the NP.

## 2 Materials and Methods

### 2.1 KI Staining and Micro‐CT of Mouse Tails

All the animal studies were approved by the Institutional Animal Care and Use Committee (IACUC) of Northeast Ohio Medical University. C57BL/6J mice were obtained from Jackson's Laboratory (stock number #000664) and housed in standard cages with ad libitum access to water and food. Male and female mice (*n* = 3–5 per experimental group) were euthanized at 12 weeks to collect the spines and tails for KI staining. Skin‐free tails were fixed in 10% neutral buffered formalin for 24 h at room temperature, washed three times in PBS, and then placed in KI staining solution for the allotted time (30 min, 2 h, 4 h). After the staining, the tissue was directly taken to the micro‐CT scanner. The tissue was washed with either 1× PBS or water or put into 50% ethanol to remove the KI stain completely and processed for histology. KI was purchased from Thermo Scientific (#BP367), and a 50% KI stock solution in water was prepared, which was further diluted to obtain 25% and 12.5% KI using milliQ water. All solutions were stored at RT in a dark environment. NaI was purchased from Thermo Scientific (#01166522), and a 25% solution was prepared in milliQ water. Mouse tail samples were stained for 30 min at room temperature. Iohexol was obtained from TCI America (#I09035G), and a 25% solution was prepared in milliQ water, and mouse tail samples were stained for 30 min at room temperature. A SkyScan 1273 micro‐CT scanner (Bruker) was used to assess the IVD morphology using a 10‐μm isotropic voxel size. Scanner settings were maintained at 90 kV of tube voltage and 88 μA of tube current through a 1 mm Al filter. Scanned projections were reconstructed in NRecon (Bruker) and analyzed using Avizo 3D Pro (Thermo Fisher). Percent X‐ray attenuation was determined using ImageJ.

### 2.2 Annular Needle Puncture Surgery (NPS) Induced IDD in Mice

IDD in mouse caudal IVDs was established by annular puncture using a 30‐gauge needle, as described in [14, 15, 16]. Briefly, 12‐week‐old C57BL/6J male and female mice (*n* = 10) were anesthetized by exposure to isoflurane and given buprenorphine after surgery for pain relief. The IVDs were located by palpation, and a 5 mm longitudinal incision was made on the skin to expose the IVD. The caudal IVDs (Ca4/5 and Ca6/7) were punctured using a 30‐gauge needle at a controlled depth of needle bevel under a stereoscope. No leakage of IVD tissue was observed after the NPS. The skin was then closed using tissue glue. Two IVDs per mouse were punctured, with adjacent IVDs used as sham controls. The pain was analyzed in the mice before and 2 weeks post‐NPS using a Pressure Application Measurement (PAM) device (Ugo Basile, Varese, Italy) as described elsewhere [17, 18, 19, 20]. Briefly, the mice were restrained by the hand, and the tail was held at a similar angle for each mouse. The PAM transducer was pressed against the tail, and a constantly increasing pressure (30 g/s) up to a maximum of 450 g was applied by the user, guided by the PAM software. Any discomfort, vocal sound, or attempt to withdraw the tail was taken as a sign of pain, and the pressure at which this occurred was recorded as a threshold. Two measurements were recorded per animal, and the withdrawal pressure was averaged. The mice were euthanized 2 weeks post‐NPS, and the tail samples were collected, fixed in 10% NBF, and stained with KI for micro‐CT scanning.

### 2.3 Decalcification of the Mouse Tail for KI Staining

NPS was performed on mouse (*n* = 5) caudal IVDs as described above. The mice were euthanized two weeks post‐NPS and tails were collected (6 IVDs per tail), fixed with 10% NBF for 24 h followed by decalcification using Immunocal (StatLab) for 5 days. The tails were washed with 1× PBS followed by staining with KI as described above. The tails were scanned using micro‐CT before decalcification; unstained and stained with KI after decalcification to confirm the complete removal of Ca and KI staining to visualize NP in the decalcified tail.

### 2.4 Histology

Reversal of KI staining was accomplished by processing the tissue through 50% alcohol as described elsewhere [21]. The tail samples were processed for histology as described previously [22, 23, 24]. Briefly, the mouse tails were dehydrated with a series of ethanol and xylene washes, followed by embedding in paraffin. The tissue was cut into 6 μm sagittal sections and stained with Hematoxylin and Eosin (H&E) for imaging. The images were captured using a slide scanner (Olympus VS120). The NPS IVDs were scored according to the MERCY scoring system to determine the degree of IDD [25].

### 2.5 Immunofluorescent (IF) Staining

As described above, KI was displaced by 50% ethanol, and tail samples were processed for histology. The tissue was cut into 6 μm sagittal sections, deparaffinized by xylene, and rehydrated for immunostaining. Immunostaining was performed as described previously [22, 26]. Briefly, antigen retrieval was performed by microwaving slides in citrate buffer for 3 min at moderate power. Slides were then washed with 1× TBS and blocked with 5% BSA for 30 min to prevent nonspecific binding, followed by incubation with β‐actin antibody (Santa Cruz Biotechnology, #sc‐47 778, 1:200) in a humidified chamber overnight at 4°C. A secondary fluorescent antibody was applied for 1 h, followed by nuclear staining with DAPI, and then mounted the coverslip with the help of anti‐fade mounting media. A confocal microscope (Leica SP8) was used to capture images.

### 2.6 Statistical Analysis

The graphical data is expressed as mean ± SD. Statistical analysis was conducted using GraphPad Prism software (Version 10). The data was tested for normality by the Shapiro–Wilk test, then analyzed using Student's *T*‐test. *p* < 0.05 was considered statistically significant.

## 3 Results

### 3.1 Optimization of IVD Staining With KI

**FIGURE 1:**
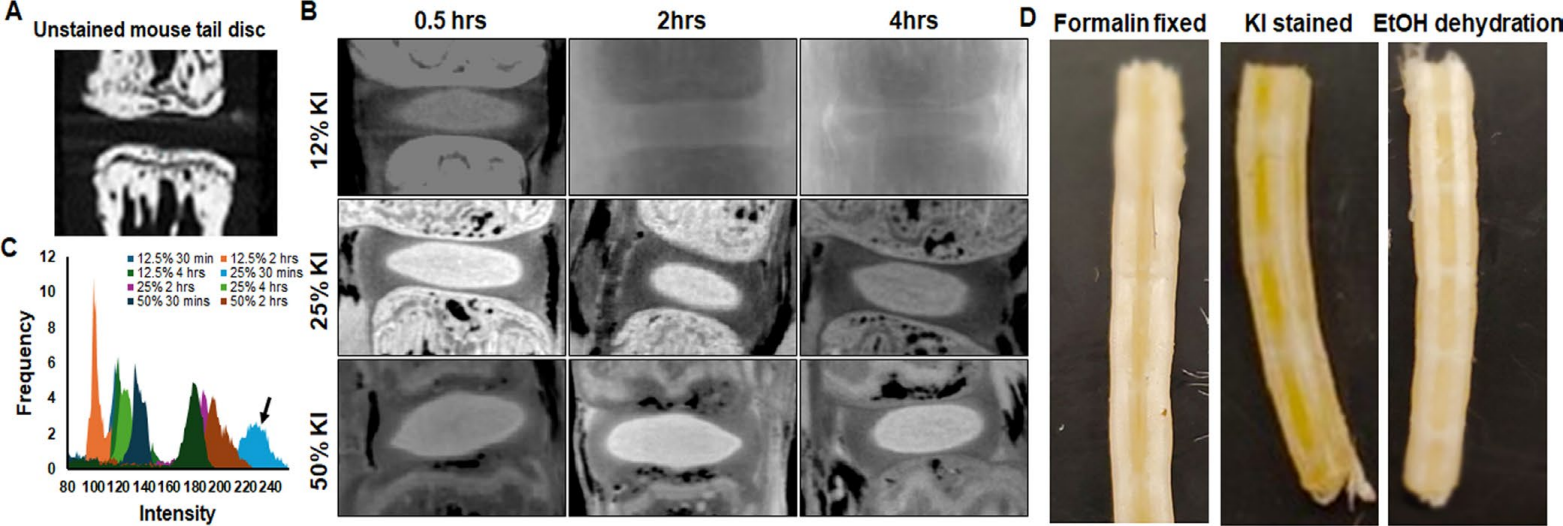
Optimization of KI staining of mouse tail IVDs. (A) Representative micro‐CT images of an unstained 12‐week‐old mouse tail IVD. (B) Mouse tails were collected from 12‐week‐old C57BL6 male and female mice (*n* = 5 per group, per time point, at least 6 IVDs per tail) and fixed in 10% NBF for 24 h followed by staining with different concentrations of KI for specified time. The samples were scanned with micro‐CT machine as described in the materials and methods. (C) KI staining intensity of NP was estimated by ImageJ. 25% KI for 30 min showed maximum attenuation intensity (indicated by black arrow). (D) Representative images of formalin fixed, KI‐stained, and EtOH dehydrated mouse tail samples showed no macroscopic damage to the tissue.

The AF, NP, and cartilage endplate tissues of IVDs do not attenuate the X‐rays and thus are invisible in micro‐CT scanning experiments (Figure 1A). To study the structure of IVD tissues, staining of tissues with contrast‐enhancing agents is a standard practice to visualize tissues that normally cannot be visualized by micro‐CT. To visualize the IVD tissues, mouse tails ranging from Ca3 to Ca9 were stained with different concentrations of KI (50%, 25%, and 12.5% w/v) for the indicated time (30 min, 2 h, and 4 h), followed by scanning by the micro‐CT machine. KI fully penetrated the IVD and provided a clear radiographic contrast to NP as early as half an hour, with almost every concentration tested in this study (Figure 1B). The NP and AF tissues were not visible using the same micro‐CT scanning parameters (Figure 1A), showing the difference between stained and unstained IVDs. We observed differential staining of AF and NP in KI‐stained IVDs with high KI uptake in the NP compared to AF. There was no macroscopic structural change observed in the tail after staining with KI (Figure 1D). In our staining optimization, we found that 25% KI in 0.5 h resulted in the best staining and radiographic contrast of mouse tail NP tissue, as shown by attenuation intensity profile data (Figure 1C), and was used in further staining studies. These results show a positive staining of NP tissue in a healthy IVD.

### 3.2 IVD Degeneration Analysis by KI Staining in an NPS Model

**FIGURE 2:**
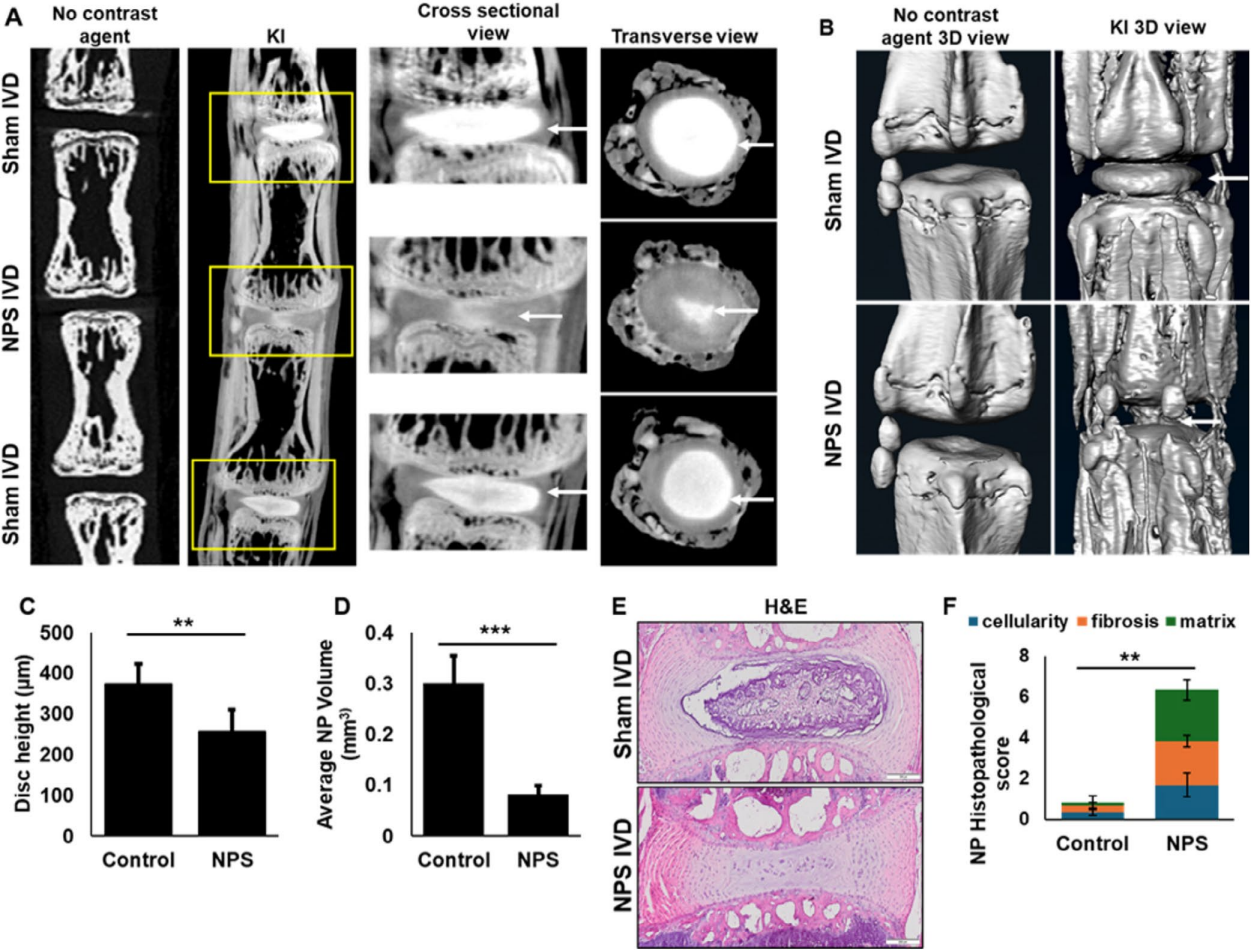
Visualization of damage to the IVD in a needle puncture surgery (NPS) model of IDD in mice. (A) 12‐week‐old C57BL/6J mice (*n* = 10) were subjected to NPS. The tails were collected 2 weeks post‐NPS (at least 6 IVDs per tail, 2 NPS and 4 sham IVDs) and processed for KI staining and micro‐CT imaging. Representative micro‐CT images of NPS and sham IVDs from unstained (no contrast agent) and KI‐stained mouse tails. The NP, indicated by the white arrow in the cross‐sectional and transverse zoomed view, showed clear damage to NP after NPS. (B) Representative 3D view of the sham and NPS IVDs showed a drastic difference in the shape and appearance of NP upon NPS. White arrows indicate the NP. (C) The IVD height in the KI‐stained control and NPS IVDs showed a significant difference (Student *t*‐test, *p* < 0.05). (D) The volume of NP in the sham and NPS IVDs showed a significant loss of NP after needle puncture (Student's *t*‐test, ***p < 0.005). (E) The tails were processed for histology and stained with H&E. Representative H&E‐stained images of control and NPS IVDs. (F) MERCY score of NP in the mouse tail showed significant IVD degeneration. (Student *t*‐test, *p* < 0.05). **p = 0.04 for cellularity, *p* = 0.01 for fibrosis, and *p* = 0.002 for matrix. These results showed that KI staining can visualize NP damage.

Previous studies using non‐ionic iodine‐based contrast agents, for example, Hexabrix, to visualize IVDs have shown a negative correlation of NP staining [12], with healthy NP stains less, and damaged NP allows high penetration with the contrasting agent. We tested our KI‐based positive staining method to determine the damage to NP in an NPS model of IDD. The NPS was performed by puncturing the mouse caudal IVD (*n* = 10) using a 30‐gauge syringe needle, as shown in the flow chart (Figure S2). The tails were harvested 2 weeks post NPS and fixed with 10% NBP prior to KI staining. Prior to euthanizing the animals, we tested pain sensitivity in the surgical IVD using the PAM device. The punctured IVDs showed increased pain sensitivity, as observed by a reduced pressure threshold (Figure S3). Non‐NPS mouse tails were used as a control to set the baseline. KI staining revealed a decrease in NP area in the NPS IVDs, compared to adjacent sham control IVDs (Figure 2A). The 3D rendering of the IVD clearly showed damage to the NP in the NPS model (Figure 2B). We also determined the IVD height (Figure 2C) and NP volume (Figure 2D) using CTAn software (Bruker), which showed a significant reduction in NPS IVD compared to sham. The observations in contrast‐enhanced micro‐CT data were tested by histology using H&E staining and displayed similar results between histology and micro‐CT (Figure 2E). We also scored damage to the NP (Figure 2F) using the MERCY scoring system [25]. These results show that KI can be used as a contrast agent to visualize damage to NP. In addition to providing contrast to NP, KI also helped visualize the osteophytes, which can be seen in 3D rendered images (Figure 2B).

### 3.3 Mouse Spine IVD Imaging With KI Staining

**FIGURE 3:**
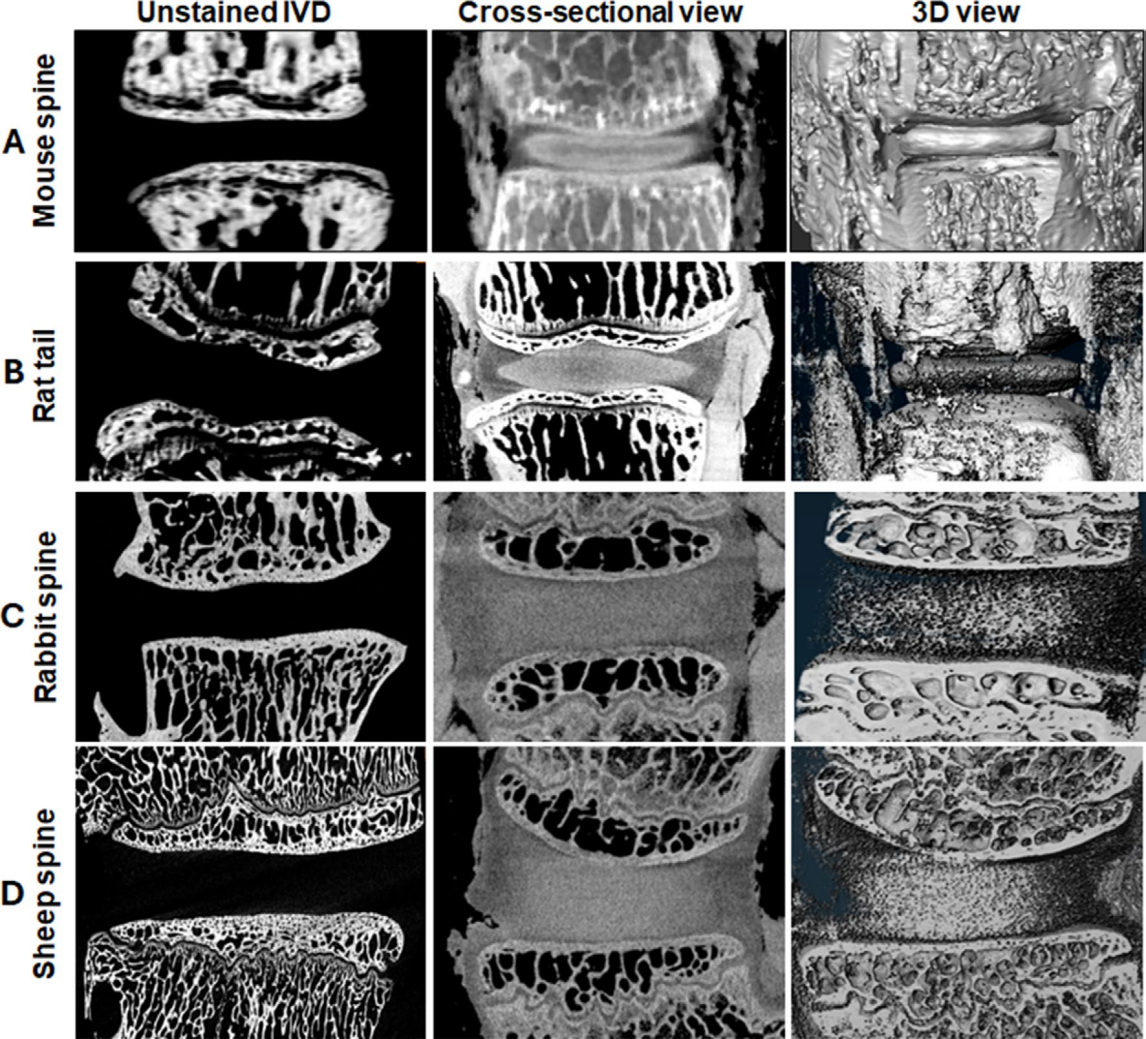
Visualization of NP in mouse spine and large animal IVDs with KI staining and micro‐CT. (A) The lumbar spine was collected from mice (*n* = 5) and incubated in KI solution for 45 min followed by imaging with a micro‐CT machine. The unstained spine was scanned as a control. (B) The rat tail (*n* = 3, at least 6 IVDs per tail, total 18 IVDs) was incubated in KI solution as above and scanned with a micro‐CT machine. The images show the staining of NP with KI in the cross‐sectional and the 3D‐rendered view. (C) Representative images of Rabbit (*n* = 7) and (D) Sheep IVDs (*n* = 5) stained with KI for 6 and 8 h, respectively.

To test KI staining for spine IVDs, we stained the mouse lumbar spine with 25% KI solution initially for 30 min, but the micro‐CT images were not as sharp as the tail images (data not shown). We then increased the staining time from 30 to 45 min. As shown in Figure 3A, the NP tissue in the spine disc was visualized by increasing the time of incubation with KI. These micro‐CT images of spine discs (cross‐sectional and 3D view) show that KI works well with IVDs from the tail and the spine.

### 3.4 KI Staining of Large Animal IVDs

We further tested KI staining to visualize NP in the IVDs of larger rodents. We stained rat tails with KI for 45 min and scanned the IVDs with micro‐CT. Interestingly, KI was found to penetrate the IVD in the rat tail, resulting in the visualization of the NP tissue (Figure 3B). We also tested KI to visualize IVDs from rabbit (6 h staining time) and sheep (8 h staining time) spines (Figure 3C,D). These results show that KI can be used as a contrast agent to enhance the visualization of the NP by micro‐CT in multiple animal models.

### 3.5 Visualization of IDD in Aging Mice With KI Staining

**FIGURE 4:**
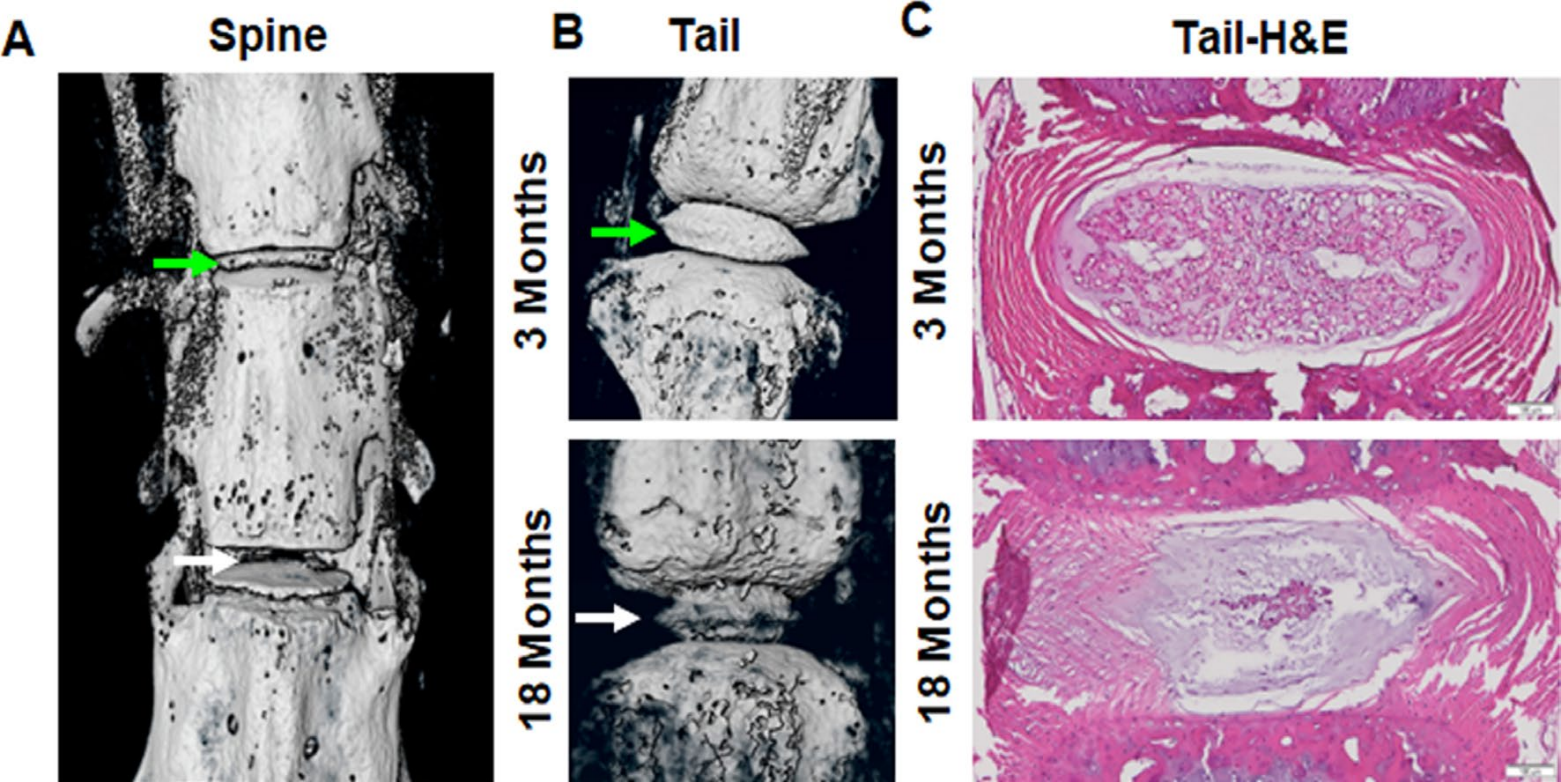
IDD analysis in aged mice with KI staining. (A) Lumbar spines were collected from 18 to 21‐month‐old mice (*n* = 5) and subjected to KI staining to visualize aging‐induced IDD. A relatively healthy IVD in the aged mouse spine is indicated by a green arrow. The white arrow indicates a complete loss of NP in the aged mouse spine. (B) Tail samples were collected from 3‐month‐old (young) and 18–21‐month‐old (aged) mice (*n* = 5 per age group, at least 6 IVDs per tail). The green arrow indicates a healthy IVD in a young mouse tail and the white arrow indicates degenerated IVD in aged mouse tail. KI staining highlighted aging‐induced IDD in both spine and tail IVDs. (C) IDD in the mouse tail was confirmed by H&E staining. The images show the degeneration of the IVD with age and also confirm that KI staining does not interfere with subsequent histological processing of the tissue.

To test if KI staining can highlight IVD damage in an aging mouse model, we collected tail and spine samples from 18 to 21‐month‐old mice (*n* = 5) for KI staining. KI staining highlighted the damaged and undamaged IVDs in the micro‐CT scan of the mouse spine (Figure 4A). KI staining also highlighted the difference between damaged and undamaged IVDs in aged mice in the micro‐CT scan (Figure 4B). Damage to aged IVDs was confirmed by H&E staining (Figure 4C). The aged IVD showed clear damage to the NP and AF compared to 3‐month‐old IVDs (Figure 4C), confirming the micro‐CT results.

### 3.6 IVD Immunofluorescent Staining Was Unaffected by Prior KI Staining

**FIGURE 5:**
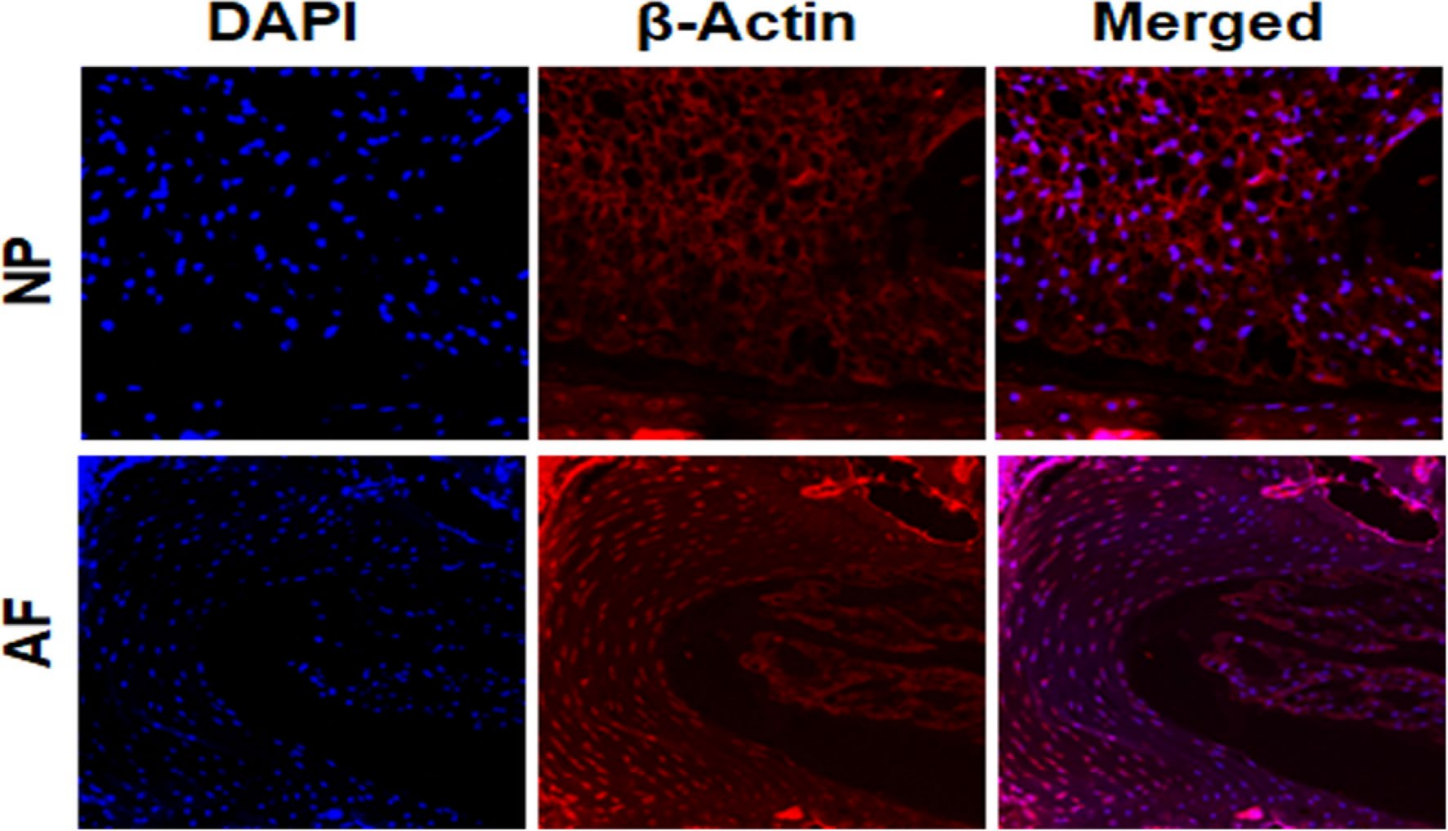
Immunostaining in IVD samples processed for KI staining. Mouse tail samples stained with KI were subsequently processed for immunostaining using β‐Actin antibody. The result showed that KI staining does not interfere with the post‐processing of the tissue for immunostaining.

After H&E staining of the IVD samples stained with KI, to test whether KI staining interferes with the immunological properties of the antigens, we subjected IVD sections to immunostaining using anti‐β‐Actin antibody. Samples were stained with β‐actin as a primary antibody, a fluorescent secondary antibody, and DAPI was used to counterstain the nuclei. β‐Actin signal was observed in both AF and NP areas of the IVD (Figure 5), indicating that antigens are preserved after KI stain.

### 3.7 Sodium Iodide (NaI) Can Provide Iodine Ions to Visualize the IVD

**FIGURE 6:**
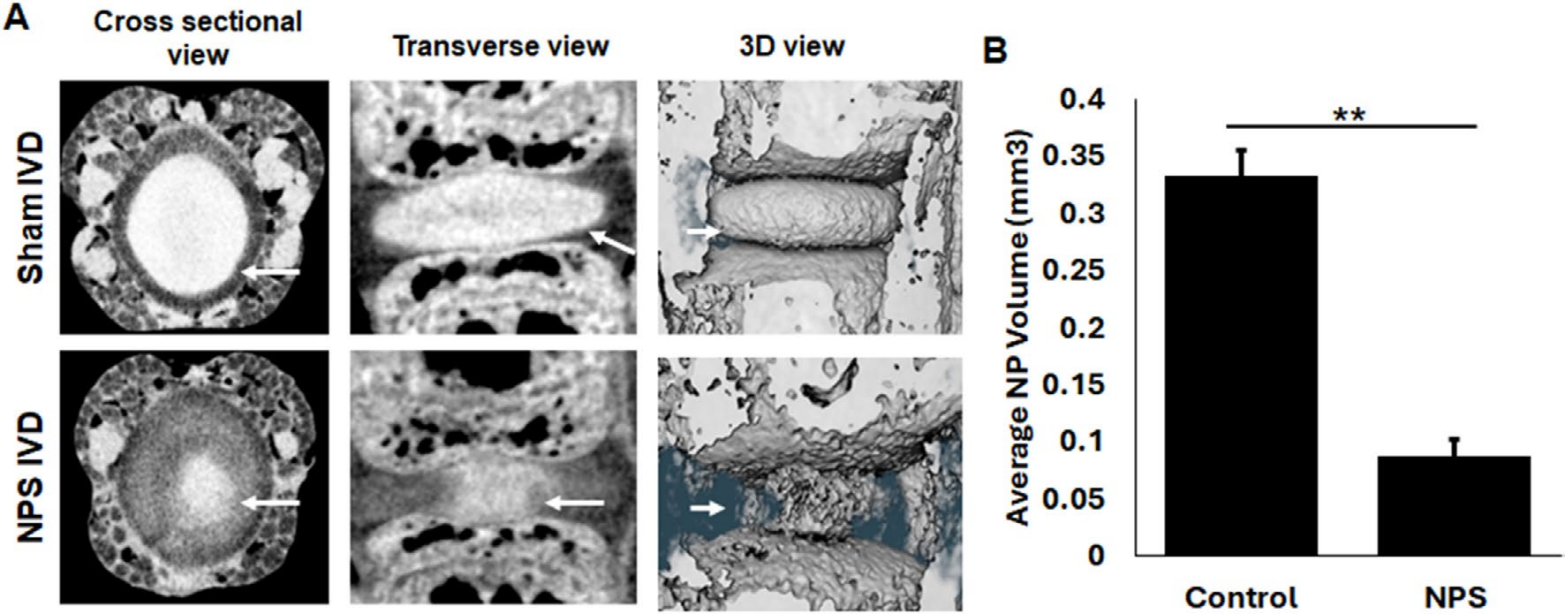
Visualization of damage to the IVD in a NPA model of IDD in mice using Sodium Iodide (NaI). (A) 12‐week‐old C57BL/6J mice (*n* = 5) were subjected to NPS. The tail samples were collected 2 weeks post‐NPS (at least 6 IVDs per tail, 2 NPS and 4 sham IVDs) and processed for NaI staining and micro‐CT imaging. Representative micro‐CT images of NPS and sham IVDs from NaI‐stained mouse tails. The NP, indicated by the white arrow in the different views, showed clear damage after NPS. Representative 3D view of the sham and NPS IVDs showed a drastic difference in the shape and appearance of NP upon NPS. (B) The volume of NP in the sham and NPS IVDs showed a significant loss of NP after needle puncture (Student's *t*‐test, **p < 0.005).

We tested whether we can use NaI in place of KI to visualize IVDs. The mouse NPS tails (*n* = 5) were stained with a 25% solution of NaI, similar to KI, and the tails were scanned by micro‐CT using the same parameters. Interestingly, similar to KI, NaI staining could provide a strong contrast to the NP (Figure 6). This suggests that it is the iodine ion in the solution that provides the contrast to the IVD, not the cation.

### 3.8 Comparison of KI With Phosphotungstic Acid (PTA) and Iohexol Staining

**FIGURE 7:**
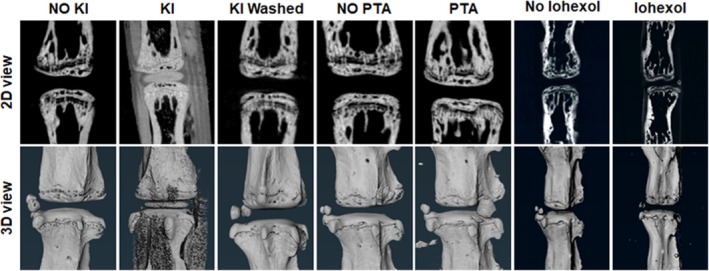
Both PTA and Iohexol failed to give contrast to NP in 30 min of staining time. We stained 12‐week‐old mouse tails (*n* = 5 per group, each tail with at least 6 IVDs, total 30 IVDs) with 25% KI, 0.06% PTA, and 25% Iohexol for 30 min followed by scanning with micro‐CT using the same parameters. Representative images from each group, before staining and after staining, show only KI visualized the NP. KI‐stained tails were washed with 1× PBS for 30 min and scanned (3rd column). This showed KI staining is reversible.

To confirm that the KI‐based staining method is fast, reliable, and reproducible compared to other conventional contrast agents, we performed staining of mouse tail IVDs (*n* = 5 tails per group, with at least 6 IVDs per tail) with KI, PTA, and Iohexol for 30 min and scanned the IVDs with micro‐CT as above. Interestingly, both PTA and Iohexol failed to provide any contrast in 30 min of staining, whereas KI staining was reproducible (Figure 7). We also showed that KI staining is reversible by washing the stained tails with 1× PBS for 30 min (Figures 7 and S4).

### 3.9 KI Stains NP in Decalcified Tail

**FIGURE 8:**
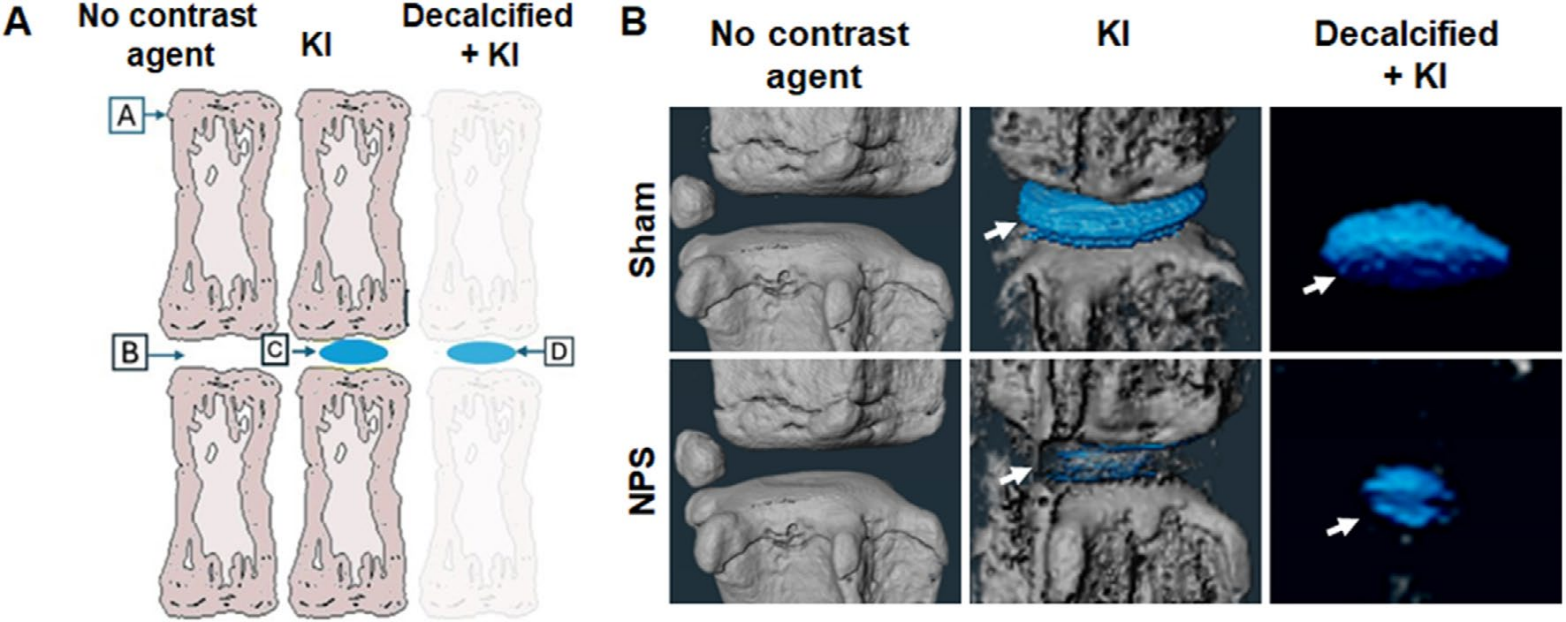
KI visualizes NP in the decalcified tail. (A) Schematic representation of IVD visualization with contrast agent. No contrast agent only shows the vertebral bone. KI contrast agent shows both, bone and NP and decalcified tissue after KI staining only shows the NP, removing the bone from the background. A = vertebral bone, B = empty IVD space (unstained IVD), C = KI‐stained IVD (with bone), D = IVD stained with KI, decalcified bone. (B) Mouse (*n* = 5, each tail with at least 6 IVDs, total 30 IVDs) caudal IVD subjected to NPS was harvested two weeks post‐surgery. The tail was scanned by micro‐CT before KI staining, after KI staining and after decalcification followed by KI staining. After decalcification, and KI staining, only the NP was visible (indicated by white arrow).

In the above approaches using KI to visualize NP, we can see the NP, but we also see the bone that requires further segmentation to analyze the changes in NP. We hypothesized that within the tested time, KI specifically stains NP, not the surrounding bone. In the unstained tissue, we only see vertebral bone; in the KI‐stained tissue, we see vertebrae and NP, and in the decalcified tissue stained with KI, we expect only to see NP (Figure 8A). To test our hypothesis, we decalcified mouse tails (*n* = 5) using immunocal and stained them with KI followed by micro‐CT scanning. As expected, the unstained tissue only showed the vertebrae; the KI‐stained tail showed both the NP and vertebrae, and the KI‐stained decalcified tail only showed the NP (Figures 8B and S5). We observed a very low staining of soft tissue, which can be removed by further optimization of the staining time and KI concentration.

## 4 Discussion

The current histological approach of evaluating changes in NP in the degenerating IVD is time‐consuming, destructive, and cannot assess the 3D spatial morphology of the NP. In addition, the semi‐quantitative scores are subjective with intra‐ and inter‐observer variations. The 3D morphometric analyses obtained through micro‐CT offer a distinct advantage over the 2D histological approach by providing an accurate volumetric assessment and eliminating the need for sample positioning and possible artifacts introduced by dehydration, embedding, and sectioning. Moreover, the samples can still be processed for histology after micro‐CT.

Contrast‐enhanced imaging of soft tissues, which otherwise have low to no X‐ray attenuation properties, is the method of choice to visualize them using CT scans. Iodine‐based contrast agents such as Lugol's solution, Hexabrix, Iohexol, and Cysto Conray II are commonly used due to their high X‐ray attenuation properties [12, 13, 27]. Here, we show that KI can be used as a contrast agent to visualize NP tissue in the IVD. The NP is a water‐rich, gelatinous structure with a high level of glycosaminoglycans (GAG), which gives it an overall negative charge. Previous studies have shown that Hexabrix staining of NP tissue is inversely proportional to the amount of GAG in the NP due to its negative charge [12]; however, we found that the negatively charged iodine ions can penetrate the NP and provide a strong contrast to it. At this stage, we do not know the mechanism of NP staining by iodine ions. Previous studies provide a negative approach to visualizing changes in NP that rely on not staining the NP. Our method provides a positive staining of NP, in which we stain the NP to visualize it through micro‐CT, and we can directly measure the changes in the NP. We also showed here that the iodine ion to provide contrast to the NP can be provided by KI or NaI. Interestingly, within the tested time of staining, we did not see any staining of the NP by either PTA or Iohexol. Overall, this study provides a fast and reproducible method for NP visualization by micro‐CT (see Table 1 for comparison).

**TABLE 1:** A summary of contrast agents with their concentration and time of staining for a comparative view.

Contrast agent	Time of staining	Concentration (mg/mL)	Sample/tissue type	References
Ioversol	24 h	175	Mouse lumbar IVD	[28]
PTA (Phosphotungstic Acid)	14 days	0.6	Bovine IVDs	[29]
I2KI (Iodine Potassium Iodide)	14 days	0.1 I2, 0.2 KI	Bovine IVDs	[29]
PMA (Phosphomolybdic Acid)	2 months	0.6	Bovine IVDs	[29]
Ioversol	Injected, live imaging (30–50 min)	350	Mouse thoracic, lumbar, coccygeal IVD	[30]
PMA (Phosphomolybdic Acid)	72 h	50	Mouse lumbar IVD	[31]
PTA (Phosphotungstic Acid)	1–5 days	100	Porcine ligaments	[8]
I2KI (Iodine Potassium Iodide)	1–5 days	33 I2, 67 KI	Porcine ligaments	[8]
PMA (Phosphomolybdic Acid)	1–5 days	100	Porcine ligaments	[8]
I2KI	Overnight to 7 weeks	Vary	Vertebrate soft tissue	[21]
Hexabrix‐320 (anionic)	24 h	128	Rat lumbar IVD	[12, 32]
Ca4+ (cationic)	24 h	30	Rat lumbar IVD	[12, 32]
Ioversol	24 h	350	Murine IVD	[33]
Hexabrix‐320 (anionic)	24 h	160	Rabbit IVD	[34]
KI (Potassium Iodide) and NaI (Sodium Iodide)	30–45 min	250	Mouse, rat, rabbit, sheep IVDs (spine and tail)	This study

This positive staining of NP in the IVD provides us with a huge opportunity to study IDD in multiple animal models, including mouse, rat, rabbit, and sheep. This method can also be optimized for other animal models and to study IDD without the dehydration of the IVD and without going for a longer incubation period (2 months incubation in [29]). Other studies require cutting and isolation of the IVD from the vertebral column or tail vertebrae [29], which poses additional challenges, may require expertise, special skills, and additional tools such as the LASER dissection tool for precise cutting to prevent any damage to the IVD. The above study also showed major macroscopic structural changes, including damage to NP and separation of AF lamellae in the isolated IVD due to fixation and longer staining time [29], while we did not see any macroscopic changes in the IVDs (Figure 1D). Histology is a vital tool for evaluating IDD in murine studies. We removed the KI from the sample and processed the tissue for histology, and did not see any adverse effect of KI on IVD histologically. Also, antigens are well preserved, and the immunostaining of the IVD sections was not affected by KI. In this method, the NP tissue was stained while present in the spine or tail, giving an added advantage to analyzing the bone in the same scan. The other advantage of this method is that we get to see the whole NP tissue in a 3D view that helps in the analysis of the entire NP in the IVD rather than looking at thin sections by histology, which shows only a small portion of the NP. The staining of KI is reversible, giving another advantage of processing the IVD samples for further histological analysis. In addition to the NP, other soft tissues around the IVD were also visualized by KI staining and can be studied. KI can also be used for IVD characterization in genetically modified mice and rats to explore any phenotype. This method can also be used to assess the health and regeneration of NP in cell transplantation therapy. The X‐ray attenuation by 25% KI‐stained NP was as high as that of bone, indicating that the imaging can be performed at lower voltage or mAmp to minimize radiation exposure while maintaining the signal‐to‐noise ratio [35]. The difference between small animals and larger animals IVDs is the time of staining. Mouse tail IVDs required 30‐min KI staining, which may be due to smaller IVD size and less surrounding soft tissues, and 45‐min KI staining time for mouse spine and rat tail IVDs, which may be due to bigger IVD size and higher amount of surrounding soft tissues. Higher time for rabbit and sheep IVDs (6 and 8 h, respectively) can be explained by the difference in size and composition of the matrix. Shorter staining time (30–45 min) did not have any effect on the macroscopic appearance of the tissues. The staining was performed with a 25% KI solution in the current study. Though it showed the IVD in larger animals, it can be further improved in future studies by optimizing the time and concentration of KI due to the difference in the size and density of the IVD's ECM in larger animals. It is important to highlight that neither KI nor NaI filled the empty spaces between the trabeculae, indicating that the staining of NP by KI or NaI is specific, but we do not yet know the mechanism. Future studies will focus on investigating the mechanism of KI staining of NP. This will help us quantify the specific component of the IVD.

In a standard micro‐CT of the spine or tail, in the early stage when there are no signs of bone remodeling, the bone appears as a background in a micro‐CT scan. In this approach, we successfully visualized the NP after decalcification of the mouse tail. This gave a better view of the NP by removing the bone background.

The limitations of this study include the lack of visualization of AF lamellar structures; however, KI staining is known to have limited revealing of tissue sub‐structures and is more suitable for larger tissues or organs in which overall anatomical structure is the subject of study [21]. PTA and PMA were shown to stain collagen‐rich AF lamellae better than KI [29]. Because all the experiments in this study were performed ex vivo on formalin‐fixed tissues, it is difficult to discuss the cytotoxic effects of KI. However, this concentration of KI is known to have cytotoxic effects and thus needs further optimization of time and concentration for in vivo experiments in future studies. It is important to note that cross‐linking of AF due to fixation by formalin may make it less permeable for KI. Future studies will include fresh, unfixed tail and spine samples to optimize KI concentration. Moreover, we were able to detect IVD staining in all the concentrations tested in this study, so we are hopeful that a lower KI concentration will work under in vivo conditions.

In summary, we show here that micro‐CT can provide high‐resolution, 3D images of NP within intact IVDs in multiple animal models. This is the first ex vivo study to show that the NP tissue can be visualized by KI or NaI staining within 30–45 min in intact IVDs. We showed here that KI‐enhanced micro‐CT can be used to analyze and quantify changes in NP structure in multiple animal models and showed that this method can be used to study changes in IVD with trauma, age, or other conditions. This is significant as the mechanism of IDD is not fully understood, and efficient assessment of NP degeneration is required for the successful treatment of IDD. The advantage of this method is that it has no macroscopic effect on the appearance of the tissue and is compatible with downstream histology and immunostaining due to its reversible nature. This study provides a platform to evaluate disease‐modifying drugs for the treatment of IDD. This approach has the potential to become a standard analysis method for comprehensive analyses of IDD and treatment efficacy.

## Conflicts of Interest

A provisional patent application has been filed for the KI‐based IVD imaging approach described in this study.

## Supporting information


**Figure S1:** Schematic representation of a healthy and degenerated intervertebral disc (IVD) structural organization. The degenerative changes in the IVD include loss of nucleus pulposus (NP), tears in annulus fibrosus (AF) lamellae, and damage to the cartilaginous end plate (CEP).
**Figure S2:** Flow chart of the study (validation or testing of the KI staining method to determine disc degeneration in needle puncture surgery (NPS) model). Briefly, the mouse tail disc was punctured using a 30‐gauge needle. The mice were sacrificed 2 weeks post‐surgery, and the IVD was analyzed by micro‐CT and histology.
**Figure S3:** Pain measurement in NPS mouse tails using a PAM device. NPS surgery was performed on the mouse caudal IVD (*n* = 5). The baseline threshold was measured before surgery, and pain sensitivity was measured 2 weeks post NPS. The numbers on the x‐axis represent the mouse IDs.
**Figure S4:** KI staining of the disc is reversible. After KI staining of the mouse tail (*n* = 5), we washed the tails with PBS for 30 min and confirmed KI removal by micro‐CT. KI staining was completely removed after PBS wash (bottom row).
**Figure S5:** KI staining after decalcification of the mouse tail. Mouse caudal IVDs (*n* = 5) were subjected to NPS for two weeks. The tails were collected and stained with KI prior to decalcification (left image) and after decalcification (right image). Decalcification successfully hid the bone in the micro‐CT scan, showing a clear view of NP.

## Data Availability

The data that support the findings of this study are available from the corresponding author upon reasonable request.
