# Supplementary material for: High‐Resolution 3‐Dimensional Micro‐CT Imaging of Intervertebral Discs Using a Novel Contrast Agent
Source: JOR Spine. 2025 Oct 7;8(4):e70125. doi: 10.1002/jsp2.70125 (PMC12502901; doi:10.1002/jsp2.70125)
Supplement: Supplementary file 1 — Figure S1: Schematic representation of a healthy and degenerated intervertebral disc (IVD) structural organization. The degenerative changes in the IVD include loss of nucleus pulposus (NP), tears in annulus fibrosus (AF) lamellae, and damage to the cartilaginous end plate (CEP). Figure S2: Flow chart of the study (validation or testing of the KI staining method to determine disc degeneration in needle puncture surgery (NPS) model). Briefly, the mouse tail disc was punctured using a 30‐gauge needle. The mice were sacrificed 2 weeks post‐surgery, and the IVD was analyzed by micro‐CT and histology. Figure S3: Pain measurement in NPS mouse tails using a PAM device. NPS surgery was performed on the mouse caudal IVD (n = 5). The baseline threshold was measured before surgery, and pain sensitivity was measured 2 weeks post NPS. The numbers on the x‐axis represent the mouse IDs. Figure S4: KI staining of the disc is reversible. After KI staining of the mouse tail (n = 5), we washed the tails with PBS for 30 min and confirmed KI removal by micro‐CT. KI staining was completely removed after PBS wash (bottom row). Figure S5: KI staining after decalcification of the mouse tail. Mouse caudal IVDs (n = 5) were subjected to NPS for two weeks. The tails were collected and stained with KI prior to decalcification (left image) and after decalcification (right image). Decalcification successfully hid the bone in the micro‐CT scan, showing a clear view of NP. [file JSP2-8-e70125-s001.docx]

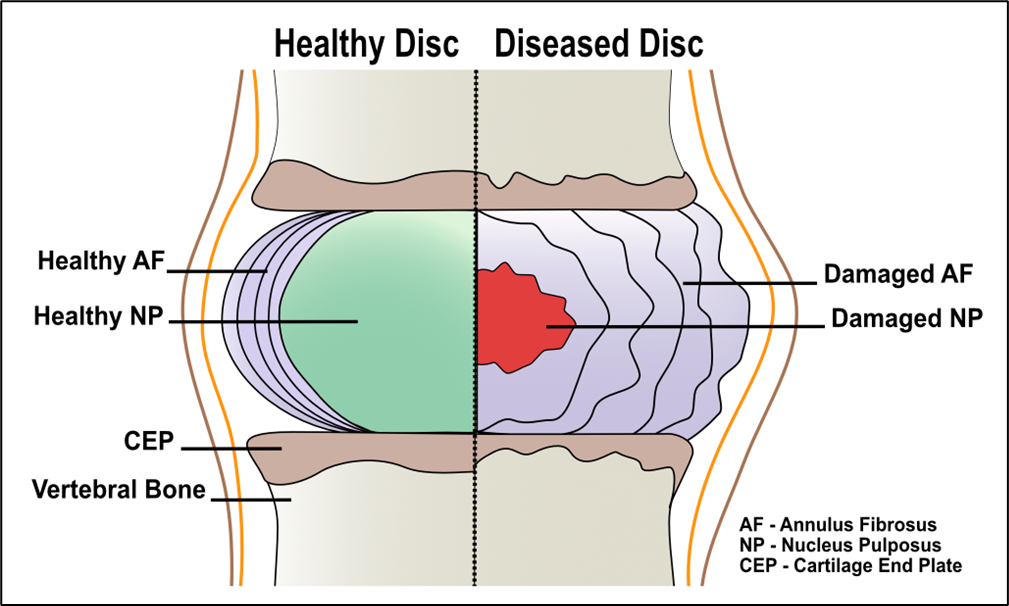


**Supplementary Figure 1:** Schematic representation of a healthy and degenerated intervertebral disc (IVD) structural organization. The degenerative changes in the IVD include loss of nucleus pulposus (NP), tears in annulus fibrosus (AF) lamellae, and damage to the cartilaginous end plate (CEP).


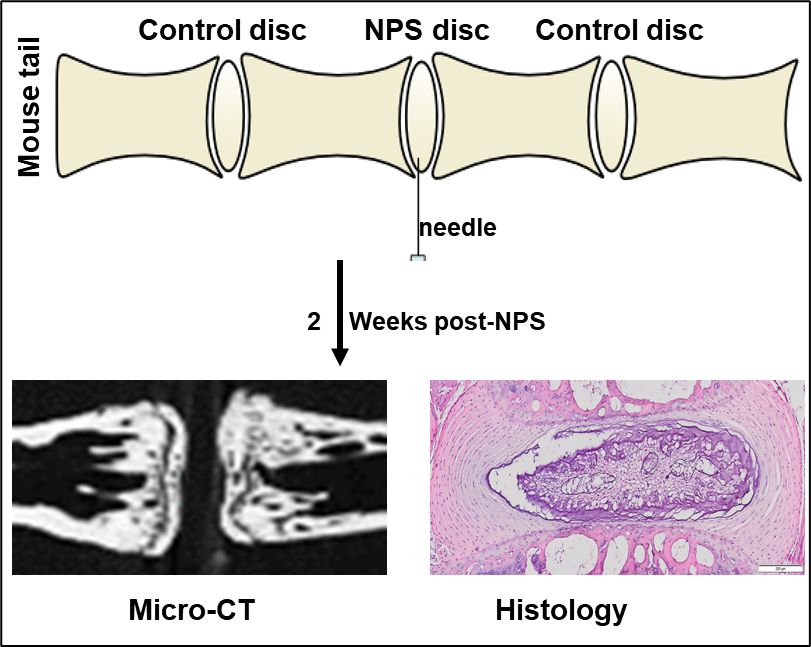


**Supplementary Figure 2:** Flow chart of the study (validation or testing of the KI staining method to determine disc degeneration in needle puncture surgery (NPS) model). Briefly, the mouse tail disc was punctured using a 30-gauge needle. The mice were sacrificed 2 weeks post-surgery, and the IVD was analyzed by micro-CT and histology.


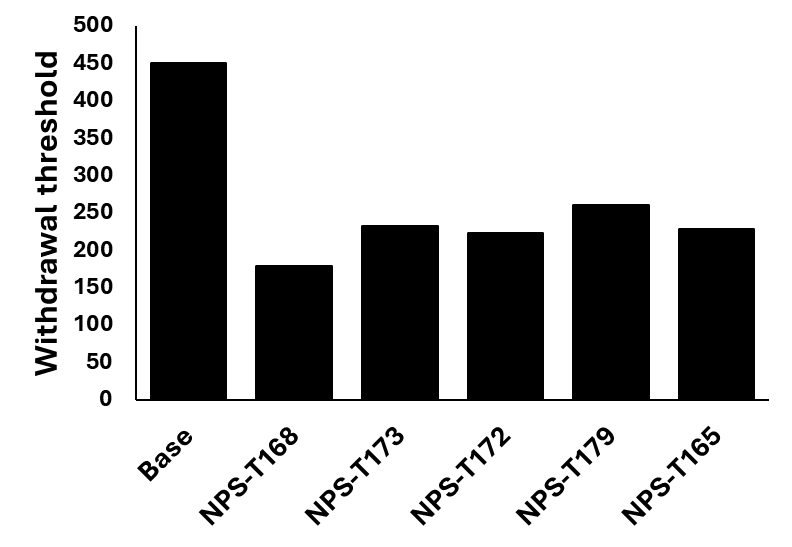


**Supplementary Figure 3: Pain measurement in NPS mouse tails using a PAM device.** NPS surgery was performed on the mouse caudal IVD (n=5). The baseline threshold was measured before surgery, and pain sensitivity was measured 2 weeks post NPS. The numbers on the x-axis represent the mouse IDs.


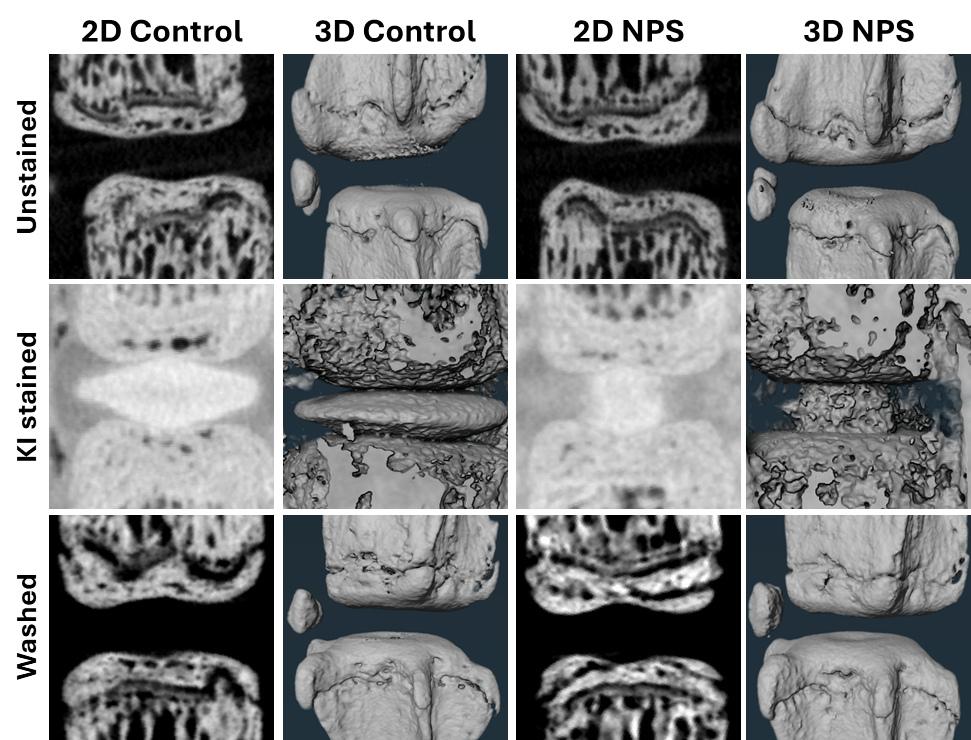


**Supplementary Figure 4: KI staining of the disc is reversible.** After KI staining of the mouse tail (n=5), we washed the tails with PBS for 30 minutes and confirmed KI removal by micro-CT. KI staining was completely removed after PBS wash (bottom row).


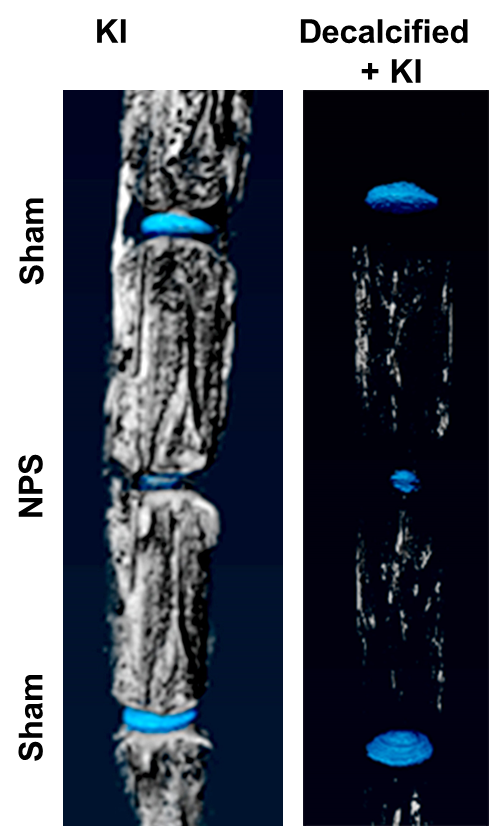


**Supplementary Figure 5: KI staining after decalcification of the mouse tail.** Mouse caudal IVDs (n=5) were subjected to NPS for two weeks. The tails were collected and stained with KI prior to decalcification (left image) and after decalcification (right image). Decalcification successfully hid the bone in the micro-CT scan, showing a clear view of NP.
